# Factors Associated With Portal and Telehealth Uptake and Use in a Minoritized, Low-Income Community: Mixed Methods Study

**DOI:** 10.2196/70146

**Published:** 2025-07-31

**Authors:** Robin T Higashi, Emily C Repasky, Antara Gupta, MinJae Lee, Catherine M DesRoches, Aimee Israel, Sandi L Pruitt

**Affiliations:** 1 Department of Social and Behavioral Sciences, Peter O'Donnell Jr School of Public Health, The University of Texas Southwestern Medical Center, 5323 Harry Hines Blvd, Dallas, TX, 75390, United States, 1 214 648-3645, 1 214-648-3934; 2 Harold C Simmons Comprehensive Cancer Center, Dallas, TX, United States; 3 The University of Texas Southwestern Medical School, Dallas, TX, United States; 4 Department of Health Data Science & Biostatistics, O'Donnell School of Public Health, The University of Texas Southwestern Medical Center, Dallas, TX, United States; 5 Department of Medicine, Harvard Medical School, Harvard University, Boston, MA, United States

**Keywords:** digital health technology, digital divide, digital inclusion, social determinants of health, health disparities, technology access, technology uptake, 21st Century Cures Act

## Abstract

**Background:**

Despite evidence that use of patient portals and telehealth is associated with many health benefits, disparities exist in awareness, adoption, and use. Understanding factors and strategies specific to underserved populations is key to achieving digital equity and better health.

**Objective:**

This study assesses portal and telehealth experiences among residents of a minoritized and lower-resource area of Dallas, Texas.

**Methods:**

Using an explanatory sequential design, we conducted surveys and semistructured interviews with English- and Spanish-speaking adults in 15 ZIP Codes surrounding a community-based clinic. We recruited participants via a patient portal, flyers, emails distributed by clinic and community partners, and in person. Surveys were offered online and on paper. We used Fisher exact tests to identify factors associated with telehealth and/or portal use. We also recruited a subsample of survey participants to expound on survey findings in semistructured interviews. Our thematic analysis assessed convergence in survey and interview findings.

**Results:**

Among 182 survey respondents, most were older (n=109, 66%; age ≥60 years), African American or Black (n=120, 65%), and female (n=142, 79%); a little more than half (n=97, 54%) had completed ≥1 telehealth appointment, and a majority (n=131, 72%) had used a patient portal at least once. Compared with those who used the portal and/or telehealth, those reporting no use of portal or telehealth were more likely to have a high school education or less (*P*<.001) or be Spanish speakers (*P*<.011). A majority, regardless of portal or telehealth use, agreed with health promotion activity survey statements like “Using the Internet for health-related activities makes me feel actively involved with my health care” (n=103, 59%) and “I find the Internet useful for monitoring my health” (n=100, 58%). In interviews with 20 individuals, most of whom were older, Black, female, and had digital technology experience, seven factors were key to increased engagement in portals and telehealth: (1) improving patient autonomy, (2) integrating digital health technology into daily life, (3) receiving recommendations from trusted individuals, (4) appreciating the value of digital health technologies, (5) enlisting the support of care partners or peers, (6) managing severe or chronic illness, and (7) accessing test results rapidly.

**Conclusions:**

This study builds on previous work by confirming and contributing insights about factors key to technology uptake and use among underserved populations. Interventions using digital health technologies should focus on these factors to promote digital and health equity and achieve better health outcomes. Future research should explore which clinical settings and contexts are most conducive to increasing digital technology uptake and use, and implementation should leverage partnerships with community groups.

## Introduction

The use of patient portals and synchronous video visits, or telehealth, has become an integral part of health care, particularly since the beginning of the COVID-19 pandemic [1-4]. Portals and telehealth have remained valuable tools for care delivery due to their many documented benefits, including convenience (reduced time, money, and burden, especially for patients living in rural areas), reduced transportation barriers, and inclusion of family members from remote areas [5,6]. Furthermore, telehealth and patient portal use are associated with increased health knowledge, patient satisfaction, and preventive services use [7-10].

Portal and telehealth use are especially valuable for people with chronic or acute illnesses such as cancer, given their high need for medical appointments, services, medications, and numerous communications with the health care system. When individuals are juggling information from multiple sources over a prolonged period, portals and telehealth serve as vital tools to organize, track, and make information and communication accessible from home or anywhere through mobile technologies. Telehealth is also especially beneficial for people with cancer, HIV, or other conditions that should avoid exposure to infection due to their compromised immune system, and because these individuals may be too physically frail or ill to attend clinic appointments [11,12].

At the same time, digital health disparities and inequities in using and accessing virtual health information remain a significant problem [13]. The digital divide has been mapped by Gallardo [14], who demonstrated using a Digital Divide Index that disparities in technological advancements parallel geographic distributions of poorer health outcomes. When populations with lower digital literacy access and skills are also the populations experiencing poorer health outcomes, these problems can be mutually exacerbating [15-17].

Low-income, minoritized populations face unique barriers to the adoption and use of telehealth and patient portals. For example, low income and low levels of education are negatively associated with uptake of digital health interventions due to low level of English literacy [18]. Additionally, language and cultural barriers present challenges to uptake of digital health interventions among minoritized and non–English-speaking populations because resources are often not tailored to fit their specific needs [19]. These challenges increase the importance of engaging local community partners to enhance the design, development, and implementation of digital health interventions [19-21]. Several studies have demonstrated the importance of user-centered design as a key strategy to addressing digital inclusion [19,22]. Partnerships with local community groups can also facilitate outreach and recruitment to specific user groups by promoting trust in the community [23]. O’Connor et al [24] in their 2016 meta-analysis of 19 qualitative studies demonstrated that telehealth utilization in underserved communities is most strongly affected by 4 key factors: improving patient autonomy, seamless integration into daily life, personal recommendations from trusted individuals, and perceived quality of the intervention.

The goal of this study was to explore attitudes toward and experiences with telehealth and the portal among a sample of residents from an underserved community in Dallas, Texas, and to explore factors associated with the use of these health technologies. We compare our findings with the key factors of O’Connor et al [24] and offer 3 additional factors that may be relevant to the development of strategies to address digital inclusion in future interventions with low-resource populations.

## Methods

### Study Design

This study used an explanatory sequential mixed methods design [25] consisting of surveys and semistructured interviews with a geographically defined sample recruited from both community and clinic settings. This study adhered to the SRQR (Standards for Reporting Qualitative Research) guidelines (the SRQR checklist is provided in Checklist 1).

### Setting

**Figure 1.**
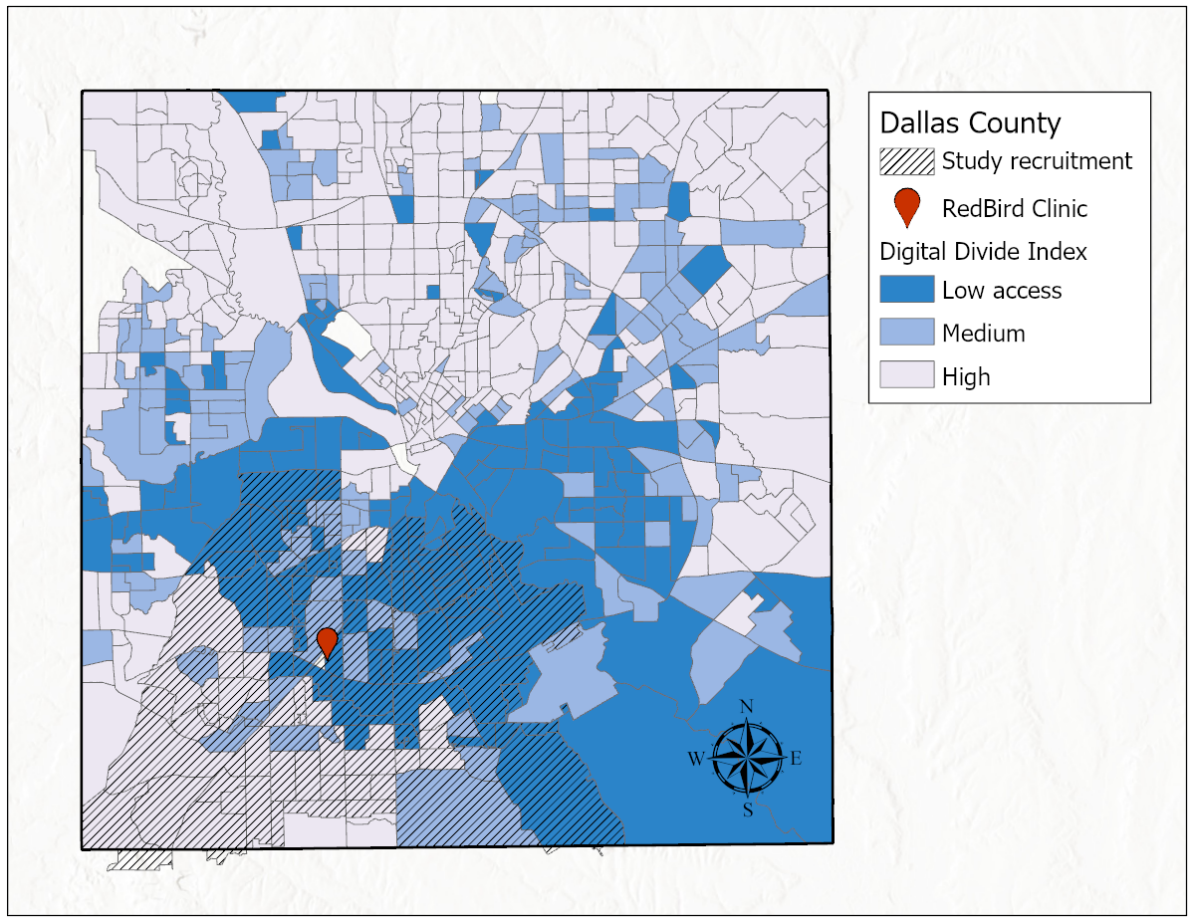
Map of Dallas County including the study recruitment area surrounding RedBird Clinic and Digital Divide Index in tertiles based on the distribution across the United States. RedBird Clinic is the community-based clinic affiliated with UT Southwestern Medical Center and the geographic center around which we based recruitment for this study. Digital Divide Index refers to a multicomponent scoring system developed by Gallardo [14] that combines socioeconomic and infrastructure elements, and “low access” reflects lowest tertile of digital access within this score.

This study was conducted with English- and Spanish-speaking individuals (age ≥18 years) and residence within the RedBird neighborhood of Dallas. We defined the “RedBird area” as comprising 15 ZIP Codes surrounding a community-based clinic affiliated with UT Southwestern Medical Center and Simmons Comprehensive Cancer Center, hereafter referred to as “RedBird Clinic.” The RedBird area in the Southern Sector of Dallas is known to be adversely affected by the digital divide, illustrated in Figure 1 [14]. Gallardo [14] calculated the Digital Divide Index by adding a composite socioeconomic score (age, poverty, lower than high school education, disability status, and internet-to-income ratio) with an infrastructure and adoption score (broadband access, computer devices, and download and/or upload speed).

### Survey Sampling and Recruitment

We recruited survey participants through the RedBird Clinic, the Simmons Comprehensive Cancer Center (hereafter “Cancer Center”), and through local community partners including faith-based organizations, nonprofit organizations, and support groups. All organization partners were located in the RedBird neighborhood from which we recruited study participants, and all were dedicated to advancing the social, economic, and/or health needs of RedBird area residents. Cancer Center participants were sampled through the patient registry, limited by those with residential addresses within the 15 Zip Code area and having completed an appointment within the past 5 years.

To capture data from participants of varying levels of digital literacy, we deployed multiple survey recruitment and data collection methods in accordance with each community partner’s preference. Ultimately, most surveys were completed either on paper or through REDCap (Research Electronic Data Capture) [26], a web-based data collection and management tool. Recruitment strategies included mailing paper surveys, sending a link for distribution in the organization’s newsletter, distributing flyers with a QR code and a phone number, and in-person recruitment. Patients of the Cancer Center who had portal accounts received a survey invitation link through the portal, while nonportal account holders received a postcard with multiple options for survey administration. In all formats, the first question of the survey explained the voluntary nature of participation, and that submission of survey responses indicated consent to participate.

### Survey Data Collection and Analysis

The survey included questions about technology access, health literacy, information seeking and literacy, and portal and telehealth exposure. Descriptive statistics were used to summarize participant characteristics and survey responses. Next, to identify factors associated with portal and telehealth use, we compared their use by demographic characteristics and by health promotion behaviors using bivariate Fisher exact tests.

The survey also contained 6 open-ended questions focusing on portal and telehealth use, preferences, and experiences. We included questions related to both the portal and telehealth, going beyond the study by O’Connor et al [24] that focused on telehealth alone. Portal use is critical to study because portals are used by a greater share of patients than telehealth, and increasingly, health care systems use the portal as the gateway to their telehealth application [27,28]. Open-ended survey text data were analyzed using axial coding techniques, and thematic findings were used to refine the semistructured interview guide [29].

### Interview Sampling and Recruitment

Based on survey findings, we created a semistructured guide to probe interview participants’ perceptions and experiences with the portal and telehealth, including factors that motivated their enrollment and use. We also added questions about the role of care partner support and experiences related to accessing test results, given emergent findings from the open-ended survey questions that these factors were associated with technology uptake and use (Multimedia Appendix 1).

We recruited interview participants from a subsample of survey completers who expressed a willingness to be contacted for follow-up interviews. We stratified interview sampling to recruit half of the participants from the survey cohort recruited through the Cancer Center and half through various RedBird area community organizations. Within each group, we prioritized recruitment of participants whose responses to the open-ended survey questions were more robust, presuming that this might correlate with greater engagement in the interviews. As we accrued participants, we monitored the demographics of our sample to ensure that we achieved diversity by gender and race or ethnicity that reflected the RedBird area population. We made up to 3 attempts to reach each participant via their preferred mode of contact. During recruitment, we also verified whether the individual had “consistent” or “mostly reliable” access to the web and/or access to a smartphone to support our focus on individuals with potential access to the portal and telehealth. That is, we chose to focus on those without device or Wi-Fi access barriers because the goal of our inquiry was to identify potential opportunities for educational interventions, such as barriers due to misperceptions, attitudes, or limited skills. Furthermore, device affordability and Wi-Fi infrastructure were improving under the Affordable Connectivity Act and broadband expansion by leading telecommunications companies.

### Interview Data Collection and Analysis

A trained qualitative interviewer (ER) conducted interviews by phone using the semistructured interview guide. Interviews lasted an average of 30 minutes (range 16‐61 minutes). Interviewers conducted rapid data analysis [30,31] during interview data collection to allow for immediate exploration of emerging patterns and insights. Rapid data analysis also serves as a tool to enhance communication among members of the interview team, facilitate monitoring effectiveness of the interview guide and determination of thematic saturation, and promote consistency of data capture.

Concurrently, we also conducted an in-depth qualitative analysis of interview transcripts. All transcript data were thematically coded by trained qualitative researchers using NVivo (Lumivero). The lead qualitative investigator (RTH) drafted an initial codebook deductively driven by concepts in the interview guide and inclusive of emergent findings extracted from the rapid analysis. Next, 2 qualitatively trained staff (ER and AG) engaged in open coding of 15% of the dataset (n=3 transcripts), iteratively refining the codes and definitions to match themes and patterns. They tested the codebook by independently coding an additional 10% of the dataset (n=2 transcripts) and then met to compare codes and calculate an interrater reliability score using Cohen κ. Because the rate of concordance exceeded 0.75, indicating substantial agreement, the remaining transcripts (n=15) were divided between the coders and analyzed independently. The research team met weekly to discuss and interpret findings. None of the qualitatively trained researchers engaged in data collection or analysis were physicians or had clinical relationships with participants.

### Ethical Considerations

All study-affiliated personnel have current certification in the ethical conduct of human subjects research on file with the University of Texas Southwestern Medical Center (UTSW)’s Human Research Protection Program. We obtained informed verbal consent for participation and audio recording in accordance with the approved study protocol from the institutional review board at UTSW (STU-2023‐0469). All audio recordings were transcribed verbatim and deidentified by a professional transcription vendor contracted with UTSW under a Health Insurance Portability and Accountability Act waiver. Participants were mailed a $30 gift card in appreciation for their participation.

## Results

### Participant Characteristics

We distributed 1262 total surveys via (1) digital methods: 646 were distributed and 64 completed, and (2) paper: 616 were distributed and 118 completed. Characteristics of the 182 total participants (182/1262, 14.4% overall response rate) did not differ by survey modality (data not shown, chi-square *P* value >.05) (Table 1). Of 182 survey participants, most were older (n=109, 66%; age ≥60 years), African American or Black (n=120, 66%), and women (n=142, 79%). Survey participants reported experience with health technology: a little more than half (97/182, 54%) had completed a telehealth appointment, and a majority (131/182, 72%) had used a patient portal.

For interviews, we contacted 59 individuals, 3 declined to participate (reason cited was “not interested”), 7 were ineligible due to lack of a Wi-Fi connection or a smartphone, and 29 did not respond to ≤3 recruitment attempts. Of the 20 semistructured interview participants, most were older (n=14, 70%; age ≥60 years) and 40% (n=8) were men. We had attempted to diversify our interview sample with individuals of varying levels of portal and telehealth experience; however, due to lack of recruitment response among portal and/or telehealth nonusers, our interview sample included a greater proportion of those with greater technology experience (Multimedia Appendix 2). Nearly all interview participants had used the portal at least once (18/20, 90%), and a majority completed at least 1 telehealth appointment (12/20, 60%).

**Table 1.** Survey (N=182) and interview (N=20) participant characteristics.

Characteristics	Survey participants	Interview participants
Age (years), n (%)		
<40	22 (13.3)	2 (10)
40‐59	35 (21.1)	4 (35)
60‐69	40 (24.1)	7 (35)
≥70	69 (41.6)	7 (35)
Unknown	16	0
Gender, n (%)		
Female	142 (78.5)	12 (60)
Male	39 (21.5)	8 (40)
Unknown	1	0
Race/ethnicity (check all that apply), n (%)		
Non-Hispanic African American or Black	120 (65.9)	11 (55)
Non-Hispanic White	28 (15.4)	6 (30)
Hispanic	23 (12.6)	2 (10)
Other	11 (6.0)	1 (5)
Education, n (%)		
High school graduate or less	51 (28.5)	6 (30)
Some college/college graduate	91 (50.8)	12 (60)
Postgraduate	37 (20.7)	2 (10)
Unknown	3	0
Language, n (%)		
English	174 (95.6)	20 (100)
Spanish	8 (4.4)	0
“Have you ever used the portal?”, n (%)		
Yes	131 (72.0)	18 (90)
No	51 (28.0)	2 (10)
“Have you ever completed a telehealth appointment?”, n (%)		
Yes	97 (53.3)	12 (60)
No	83 (46.4)	8 (40)
Unknown	2	0
Portal and telehealth use combined, n (%)		
Used both the portal and telehealth	91 (50.0)	12 (60)
Used the portal only	40 (22.0)	3 (15)
Used telehealth only	6 (3.3)	3 (15)
Used neither the portal nor telehealth	43 (23.6)	2 (10)
Used portal and unknown telehealth use	2	0
Recruitment method, n (%)		
Cancer Center	98 (53.8)	10 (50)
Community outreach	84 (46.2)	10 (50)

### Survey Findings

As shown in Table 2, more than half of our sample used both the portal and telehealth (91/180, 51%), and nearly a quarter (43/180, 24%) reported not using either technology; the remainder used 1 technology or the other. We combined those using only 1 technology or the other due to a very small number of patients reporting use of telehealth but no portal use (n=6). Compared with technology users, those reporting no use of portal or telehealth had a lower level of education (*P*<.001) or were more likely to be Spanish speaking (*P*<.011).

Table 3 illustrates frequency of endorsement of each statement about using the web for health promotion activities. The most commonly endorsed statement was “I can use my computer or mobile phone to communicate about my health needs” (81.5%). The percent agreeing to the statements varied statistically significantly by portal and/or telehealth (*P*<.001), with greater agreement among those using portal and telehealth use.

**Table 2.** Demographic characteristics of survey participants by ever use of the portal and/or telehealth^a^.

Characteristics		Categories of participants by ever use of the portal and/or telehealth^a^						
		Both, 91 (51%)^b^	One technology, 46 (26%)^b^	Neither, 43 (24%)^b^	Overall (N=180)	*P* value^c^	
Age (years), n (%)						.71	
<40		13 (15.3)	6 (14.3)	3 (8.1)	22 (13.4)		
40‐59		15 (17.6)	12 (28.6)	7 (18.9)	34 (20.7)		
60‐69		21 (24.7)	10 (23.8)	9 (24.3)	40 (24.4)		
≥70		36 (42.4)	14 (33.3)	18 (48.6)	68 (41.5)		
Unknown		6	4	6	16		
Gender, n (%)						.22	
Female		72 (79.1)	39 (84.8)	29 (69.0)	140 (78.2)		
Male		19 (20.9)	7 (15.2)	13 (31.0)	39 (21.8)		
Unknown		0	0	1	1		
Race/ethnicity, n (%)						.095	
Non-Hispanic African American or Black		56 (61.5)	32 (69.6)	31 (72.1)	119 (66.1)		
Non-Hispanic White		21 (23.1)	5 (10.9)	2 (4.7)	28 (15.6)		
Hispanic		9 (9.9)	5 (10.9)	8 (18.6)	22 (12.2)		
Other		5 (5.5)	4 (8.7)	2 (4.7)	11 (6.1)		
Education, n (%)						<.001	
High school graduate or less		9 (10.0)	11 (24.4)	29 (69.0)	49 (27.7)		
Some college/college graduate		55 (61.1)	25 (55.6)	11 (26.2)	91 (51.4)		
Postgraduate		26 (28.9)	9 (20.0)	2 (4.8)	37 (20.9)		
Unknown		1	1	1	3		
Survey language, n (%)						.011	
English		89 (97.8)	46 (100.0)	38 (88.4)	173 (96.1)		
Spanish		2 (2.2)	0 (0.0)	5 (11.6)	7 (3.9)		
Recruitment location, n (%)						.43	
Community		39 (42.9)	25 (54.3)	19 (44.2)	83 (46.1)		
Simmons		52 (57.1)	21 (45.7)	24 (55.8)	97 (53.9)		

a Two individuals who had unknown telehealth use were not included in this analysis.

b n (%).

c Fisher exact test.

**Table 3.** Survey responses regarding health promotion activity by ever use of the portal and/or telehealth.

	Categories of participants by ever use of the portal and/or telehealth				
Variable	Both, 91 (51%)^a^	One technology, 46 (26%)^a^	Neither, 43 (24%)^a^	Overall (N=180)	*P* value^b^
Overall technology access: I can use my computer or mobile phone to communicate about my health needs, n (%)					<.001
Agree	85 (93.4)	36 (78.3)	24 (58.5)	145 (81.5)	
Disagree	4 (4.4)	6 (13.0)	13 (31.7)	23 (12.9)	
Neither agree nor disagree	2 (2.2)	4 (8.7)	4 (9.8)	10 (5.6)	
Unknown	0	0	2	2	
Health literacy: I can use the internet to learn about health topics that are relevant to me, n (%)					<.001
Agree	83 (91.2)	37 (82.2)	24 (58.5)	144 (81.4)	
Disagree	5 (5.5)	3 (6.7)	13 (31.7)	21 (11.9)	
Neither agree nor disagree	3 (3.3)	5 (11.1)	4 (9.8)	12 (6.8)	
Unknown	0	1	2	3	
Improving health autonomy: I know how to find helpful health resources on the internet, n (%)					<.001
Agree	86 (94.5)	36 (80.0)	19 (46.3)	141 (79.7)	
Disagree	4 (4.4)	4 (8.9)	17 (41.5)	25 (14.1)	
Neither agree nor disagree	1 (1.1)	5 (11.1)	5 (12.2)	11 (6.2)	
Unknown	0	1	2	3	
Appreciating the value of digital health technology: I can use the internet to learn how to manage my health in a positive way, n (%)					<.001
Agree	71 (78.0)	31 (70.5)	19 (46.3)	121 (68.8)	
Disagree	8 (8.8)	7 (15.9)	17 (41.5)	32 (18.2)	
Neither agree nor disagree	12 (13.2)	6 (13.6)	5 (12.2)	23 (13.1)	
Unknown	0	2	2	4	
Health literacy: I can summarize basic health information from the internet in my own words, n (%)					<.001
Agree	74 (81.3)	25 (54.3)	13 (33.3)	112 (63.6)	
Disagree	7 (7.7)	11 (23.9)	15 (38.5)	33 (18.8)	
Neither agree nor disagree	10 (11.0)	10 (21.7)	11 (28.2)	31 (17.6)	
Unknown	0	0	4	4	
Confidence using technology: The internet improves my communication with health professionals, n (%)					<.001
Agree	68 (74.7)	22 (48.9)	16 (39.0)	106 (59.9)	
Disagree	8 (8.8)	7 (15.6)	19 (46.3)	34 (19.2)	
Neither agree nor disagree	15 (16.5)	16 (35.6)	6 (14.6)	37 (20.9)	
Unknown	0	1	2	3	
Integrating use of digital health technology into one’s daily life: I can use information on the internet to make informed decisions about my health, n (%)					<.001
Agree	64 (70.3)	23 (51.1)	18 (43.9)	105 (59.3)	
Disagree	9 (9.9)	10 (22.2)	17 (41.5)	36 (20.3)	
Neither agree nor disagree	18 (19.8)	12 (26.7)	6 (14.6)	36 (20.3)	
Unknown	0	1	2	3	
Confidence using technology: Using the internet for health-related activities makes me feel actively involved with my health care, n (%)					<.001
Agree	63 (69.2)	25 (55.6)	15 (37.5)	103 (58.5)	
Disagree	9 (9.9)	8 (17.8)	17 (42.5)	34 (19.3)	
Neither agree nor disagree	19 (20.9)	12 (26.7)	8 (20.0)	39 (22.2)	
Unknown	0	1	3	4	
Improving health autonomy: I find the internet useful for monitoring my health, n (%)					<.001
Agree	66 (72.5)	25 (56.8)	9 (22.5)	100 (57.1)	
Disagree	7 (7.7)	8 (18.2)	22 (55.0)	37 (21.1)	
Neither agree nor disagree	18 (19.8)	11 (25.0)	9 (22.5)	38 (21.7)	
Unknown	0	2	3	5	

a n (%).

b Fisher exact test.

### Interview Findings

We found evidence supporting 4 key factors by O’Connor et al [24]: improving individuals’ health autonomy, integrating use of digital health technology into individuals’ daily lives, obtaining recommendations from trusted individuals, and appreciating the value of digital health technology.

#### Improving Individuals’ Health Autonomy

Many participants reported that using technology, such as the patient portal, empowered them with easy access to information about their health and in a way that was understandable to them.

*Well, I’d probably describe it as a good way to stay up on your personal health without being worried about wasting time or having to wait for pathology. If you have questions, you can always message the clinic or there is some place within MyChart, which explains certain texts and what the results mean. It’s really pretty easy for you to stay up on what’s going on with you personally*.

[P04, experience with both the portal and telehealth]

Using the portal also facilitated participants’ self-efficacy and the belief in their ability to improve their health.

*Well, I like MyChart because it makes you focus on your health more better. [It lets] you know what’s wrong with you or [it lets] you know that you need to work on fixing this. It’s not too late to fix it.*

[P10, no telehealth or direct portal experience but a care partner helps her use the portal]

The portal also helped patients take a more active role in making decisions about their health and in leading conversations with their health care providers.

*I would say using MyChart is about being pro-help-for-yourself and knowing how to say, hey, this is what’s going on with me. I can make my own healthy choices and also confirm with my doctor, my nurse practitioner, of what these choices are and what they think or suggest about it. So MyChart is a tool for a person to make healthy choices for themselves and stay informed*.

[P17, experience with both the portal and telehealth]

#### Integrating Use of Digital Health Technology Into Individuals’ Daily Lives

Many participants found the portal to be a helpful tool to access all their health information, especially given the fact that they have multiple medications.

*There have been many times where I need to go back and look...like say, for example, I call Medco and say, ‘I need to go over my medications.’ I don’t have them written down anywhere, but I can go to my last visit and look at all my medications that are listed.... Or if I’ve been given special instructions, I’ll go back to the visit summary and look at whatever the details were for that particular visit*.

[P15, experience with both the portal and telehealth]

Participants liked the ease of access through their cell phone, presumably a technology with which they were already familiar.

*I think it’s actually pretty cool that you can get into medical charts through your phone and be able to set appointments*.

[P13, experience with the portal only]

#### Obtaining Recommendations From Trusted Individuals

Many participants who enrolled in the portal reported first learning about it from a medical provider or clinical staff.

*[At the] doctor’s office... They explained how it worked and that I could keep up with my appointments and my drugs and everything–pertinent information*.

[P05, experience with both the portal and telehealth]

Establishing a relationship with the provider first was the key to a successful portal interaction for 1 patient.

*Once you visit your doctor, you know who he or she is and you’re comfortable with them, I don’t think that’s much of a problem, ‘cause you have a relationship*.

[P11, experience with the portal only]

#### Appreciating the Value of Digital Health Technology

For 1 participant, the portal offered a more rapid and effective solution to a billing problem than troubleshooting by telephone.

*I was getting a bill for some treatment... I called the billing department, and they gave me a voicemail and they just put me on hold. It don’t work [sic]. But then I went into my MyChart and went to the billing link and left a message stating that I shouldn’t have been billed for it... They answered it and removed that and sent me an email back in MyChart saying that they had been verified that I did have an insurance coverage and I shouldn’t have been billed*.

[P09, experience with the portal only]

Some participants also valued the portal’s lesser-known features, such as appointment reminders and the option to view appointment locations on a map, which streamlined navigation across an expansive medical campus.

*Well, for one thing, it keeps me up to date on when I have appointments and also with all the different buildings as to which building it’s in and where is it on the map... That’s very helpful to me... Yeah, you can see the schematic of where all the buildings are that are running around the [location]*.

[P02, experience with both the portal and telehealth]

Multiple participants noted that the portal offers an efficient and dependable channel for communicating with their care team. They valued the rapid response via the portal, especially in comparison with phone interactions.

*It’s an easy way to communicate with the doctor because sometimes [when] you call on your phone, you’ll be on that phone, nobody will answer. Or they’ll answer, they keep you on hold [so long] that you get frustrated, and you hang up the phone. With MyChart, when you send the email to them, they will respond. It may take about five or six hours or two hours of work, but they will still reply the same day to let you know what is going on and that will give you hope that you’ll get it*.

[P01, experience with both the portal and telehealth]

In addition, participants in this study uniquely reported 3 additional key factors that encouraged uptake and use of the portal and telehealth: enlisting support from care partners or peers, managing severe or chronic illness, and accessing test results rapidly.

#### Enlisting Support From Care Partners or Peers

Several participants relied on family members to use the portal and/or telehealth.

*My daughter–she works at a breast cancer clinic so she pretty much know how to do all that [portal and telehealth] at home...she will tell me my blood pressure was very high. I would tell her, ‘yeah, the doctor told me I need to work on my blood pressure.’ And then she looked at it again and she said, ‘Oh, your blood pressure has went down.’ It just lets you know. It show you what’s going on*.

[P10, no direct telehealth or portal experience but uses portal through a care partner]

Other participants spoke of being a care partner for family members or friends and highlighted how technology facilitated management of the patient’s medical needs.

*Well...it helps me, with him being in a wheelchair, it saves me time to be able to help him and to get all the things done that he needs as far as his physical therapy and all of his appointments in line and all of his blood work that lets me know that he’s doing okay from day to day*.

[P13, personal experience with the portal and care partner for her husband to use the portal]

#### Managing Severe or Chronic Illness

Several participants who used the portal emphasized its value when coordinating care across multiple providers.

*I have become more active in [the portal] as the number of doctors have increased. I rely on it more and more*.

[P15, experience with both the portal and telehealth]

Others described how they used the portal to manage their chronic illness, such as the following participant who had kidney disease.

*They checked me and they made an appointment and it’s all written on [the portal]. I look into my test results. And if I forget something I’ll just go back and look. I double check on my A1C or what’s my potassium because they say it’s kind of high, and now it went down. So it’s good. I can always check back*.

[P08, experience with both the portal and telehealth]

Four individuals recruited from the Cancer Center specifically mentioned that they joined the portal when they were diagnosed with cancer and started cancer treatment. One said,

*I’m not sure exactly when I started, but I had anti-cancer surgery, and I was recuperating after the stay at the hospital and [the portal] was a good way to keep up with what was going on*.

[P02, experience with both the portal and telehealth]

Another participant recognized that telehealth offered a convenient alternative to in-person visits, especially during times of acute illness or frailty.

*If you’re feeling really, really sick and you just really can’t get out, that’s very helpful that if you need to stay in bed and talk to the doctor, you can do that*.

[P15, experience with both the portal and telehealth]

#### Accessing Test Results Rapidly

Participants from both the Cancer Center and the community cohorts generally liked the portal’s facilitation of faster access to test results compared with formerly having to rely on waiting for results to be communicated via the telephone.

*I like that it don’t take a week to hear back from the doctor. Within the day or so, the doctors get back with you... it is not a hassle of having to wait on the phone or wait for the doctor to call me or get an email*.

[P13, experience with the portal only]

*I wouldn’t have known the results until probably my next appointment. But I went on MyChart and I found out what it was, so I didn’t have to wait until my next appointment with the doctor to get the results*.

[P18, experience with both the portal and telehealth]

Many people felt that waiting for the clinician to contact them with the results was more anxiety-provoking than receiving the results, even if they might be negative.

*I get this anxiety like, ‘Damn, what’s the result?’ I got thyroid [issues] so I’m like, ‘Is it good?’ I like to get mine fast…. If you want to know, then at the end of the day, that’s the whole point of having MyChart, because you want to know*.

[P16, experience with both the portal and telehealth]

At the same time, 1 participant acknowledged that, in some circumstances, receiving one’s results before a doctor had a chance to speak with them could cause more anxiety or a potentially negative experience if the test result was life-threatening.

*I’m in the last [cancer] stage. I wouldn’t want to find that out through MyChart. I would rather through a phone call… because I can’t talk to MyChart*.

[P18, experience with both the portal and telehealth]

## Discussion

### Principal Findings

In this iterative, mixed methods study, we describe attitudes toward and experiences with telehealth and the portal among a minoritized population in Dallas, Texas. We identify 7 factors that are key to the uptake and use of digital health technologies. Four of these factors had previously been identified by the meta-analysis of qualitative studies by O’Connor et al [24]: improving health autonomy, integrating digital health technology into individuals’ daily lives, receiving technology recommendations from trusted individuals, and appreciating the value of digital health technologies. The 3 additional factors we identified in this study—enlisting support from care partners, managing severe or chronic illness, and accessing test results rapidly—are valuable contributions because they provide insight into potential intervention opportunities among specific populations and clinical settings. Survey findings also align with the factors by O’Connor et al [24] that are key to telehealth utilization in underserved populations (improving health autonomy, integrating use of digital health technology into one’s daily life, and appreciating the value of digital health technology), as well as other factors we know from the literature to be relevant to successful engagement with the portal or telehealth (eg, health literacy, confidence using technology, and overall technology access). Our finding that language and level of education are associated with nonusers of the portal and telehealth underscores the importance of making the portal and telehealth accessible to populations that are Spanish-speaking and, among English speakers, with lower literacy levels. Notably, although a Spanish version of the patient portal is available in some health care systems, multiple challenges have dampened enthusiasm, uptake, and use of it [32].

Support from care partners has been described as a key facilitator for technology access among other populations, especially older individuals and those with chronic illness or disabilities [33,34]. To our knowledge, this is the first study to qualitatively describe care partner support as a key facilitator of digital health technology engagement among minoritized populations. Prior work suggests that care partners value having access to patient information and report benefits related to greater understanding of medications and follow-up steps and increased feelings of control [35]. Care partner assistance with patient portals is more common when patients are older, have limited English proficiency or lower overall literacy, and greater illness severity [36]. While our sample was older, our findings around care partner support as a facilitator suggest that care partners may also play a more prominent role among lower-resource, minoritized populations.

Second, our finding about the value placed on rapid access to test results has far-ranging implications across clinical settings and populations. Participants ranked rapid access to this information as a key to motivating portal use. For example, individuals recruited from the Cancer Center expressed mostly positive and some negative opinions of rapid access to test results. Specifically, a few participants felt that patients should not be given a cancer diagnosis via the portal, but that access to laboratory results and appointments was very important. At the same time, individuals recruited from the community (who presumably were less likely to be in active cancer treatment) also felt strongly that rapid access to appointments, medications, and the opportunity to communicate with their providers was a strong motivator for portal use. While we did not ask about community participants’ medical conditions, this finding may reflect higher rates of chronic illness in lower-resource populations, such as diabetes and hypertension, which also involve regular appointments and coordination across multiple providers.

The 21st Century Cures Act [37], which functionally required that institutions immediately make available to patients, on request, information in the electronic health record, has spurred debate since it was passed in 2021 [38,39]. Most health care organizations choose to meet this regulatory requirement by making results automatically viewable on the patient portal as soon as they are available in electronic health records, meaning patients are often able to see results before speaking with a clinician. Consequently, patients and families living with cancer will likely receive “nonnormal” results with some frequency as they use the portal to review data, laboratory test results, and imaging. There is controversy about providing patients with immediate access to test results in part because this information can be conveyed prior to the patient speaking with their clinician [38,40]. The potential positive and negative impacts of immediate test result access are likely to vary immensely across individuals in both patient and clinician groups [41,42]. Health care organizations may wish to provide additional education and support to patients and care partners, particularly around how to configure portal settings to either receive or mute new test result notifications. Our findings showing some complex preferences about information provision among our participants suggest that a “one-size-fits-all” approach will not be sufficient.

Digital inclusion, or “[ensuring] that all individuals and communities, including the most disadvantaged, have access to and use of digital tools,” [43] must be emphasized in order to overcome the existing digital divide [13]. With tools such as Gallego’s Digital Divide Index and the Centers for Disease Control and Prevention’s social vulnerability index [44], among others, social epidemiologists have provided ample evidence of where behavioral interventionists should implement more intensive interventions to maximize use of resources to improve health outcomes [45]. Additionally, intervention strategies shown to be effective in addressing digital health equity have emphasized user design (eg, compatibility with existing devices and culturally appropriate content), supportive infrastructure (eg, assistance with devices and connectivity), and educational support (ie, from professionals, family, or friends) [46]. Our findings of the importance of care partners, rapid access to test results, and coordinated care for individuals with more complex disease contribute to this shared understanding of what, where, and to whom digital health equity interventions should be applied. And as a next step, using an implementation science framework such as Glasgow’s RE-AIM (1999) may help us optimize how the intervention is applied [47].

Finally, it is important to recognize that providers and health care systems contribute to the persistence of digital health disparities. Multiple studies using data from the Health Information National Trends Survey have documented bias in provider recommendations for portal and telehealth use, with greater encouragement directed toward patients who are female, White, and college-educated [48-50]. Other Health Information National Trends Survey–based studies have found that provider recommendation is a key driver of telehealth use [51] and that adults with limited English proficiency are more likely than those proficient in English to seek health information from professionals [52]. Notably, 1 study found that Spanish-preferring participants placed greater trust in information from government agencies and often turned to web-based videos for health-related information [52]. These findings illustrate the importance of educating providers and health care institutions about how provider bias can exacerbate the digital divide in health care access and disseminating reliable health information through multiple channels.

Although significant progress has been achieved in strengthening digital health access and use among underserved communities [53], several barriers remain. These include lower educational attainment [54], lack of awareness of the benefits of digital health technologies [55-57], mistrust in the health care system evidenced as concern for data privacy [58,59], and ambivalence toward technology [60]. These barriers often exacerbate existing health disparities that affect the same minoritized populations with multiple chronic conditions, who stand to benefit greatly from portal and telehealth access. Future implementation strategies to address the persistent digital divide will need to provide more personalized options, be supported by robust financial and human resources, and include trust-building with cultural minority communities [60].

### Strengths and Limitations

With only 20 interviews, in a mostly older female Non-Hispanic Black or African American population, it is not possible to generalize these findings broadly. We also chose to focus our interviews on individuals who did not have barriers in access to Wi-Fi or smartphone technology in order to delve more deeply into potential intervention opportunities that could be addressed through education versus systemic infrastructure barriers over which we had limited control. While the infrastructure for Wi-Fi is increasing, and cellular phone ownership exceeds 90%, even among low-income populations [61], this sampling strategy resulted in the exclusion of the perspectives of a more diverse range of participants, which limits generalizability and precludes a more comprehensive understanding of the issues. Additionally, while we identified care partners as an important facilitator and explored this topic in depth in interviews, we missed an opportunity to obtain quantifiable data on care partner demographics and technology-related descriptors of individuals acting as care partners by not including such questions in the survey. This would have enabled us to contribute to the literature on the demographics, technology-related skills, and attitudes of people who use technology as care partners versus those who need care partner assistance. Understanding the relationship between care providers and receivers may facilitate conceptualization of new hypotheses and strategies for interventions.

However, our study has several strengths. Our finding that the uptake and use of technology may be related to chronicity and severity of illness merits attention. While perhaps those with a more life-threatening condition may be more acutely motivated to engage in their health care, findings from this study suggest that those with chronic conditions such as diabetes, certain cancers, and hypertension may also be persuaded to use the portal to monitor their test results. Also, our study contributes uniquely to the literature by focusing both quantitatively and qualitatively on the perspectives of a multiracial, minoritized population toward digital health technologies. Our finding that language and level of education are associated with lower portal and telehealth use is useful for informing future intervention efforts. Finally, future research may be enhanced by examining behavioral outcomes, which may reveal insights regarding the possible associations between digital health interventions, health care utilization, and patient outcomes.

### Conclusions

We contribute evidence that seven key factors are relevant to the development of strategies to promote digital inclusion in future interventions for underserved populations: (1) improving patient autonomy, (2) integrating digital health technology into daily life, (3) receiving recommendations from trusted individuals, (4) appreciating the value of the technology [24], (5) enlisting support from care partners, (6) managing severe or chronic illness, and (7) accessing test results rapidly. Future studies should focus on making the portal and telehealth more accessible to English-speaking populations of lower educational attainment and Spanish speakers and should address how different clinical settings and health conditions affect the uptake of the patient portal and telehealth.

## Supplementary material

10.2196/70146 Multimedia Appendix 1 Interview guide.

10.2196/70146 Multimedia Appendix 2 Interview recruitment CONSORT (Consolidated Standards of Reporting Trials) diagram.

10.2196/70146 Checklist 1 SRQR (Standards for Reporting Qualitative Research) checklist.

## Abbreviations

REDCap: Research Electronic Data Capture
SRQR: Standards for Reporting Qualitative Research
UTSW: University of Texas Southwestern Medical Center
