# Supplementary material for: Factors Associated With Portal and Telehealth Uptake and Use in a Minoritized, Low-Income Community: Mixed Methods Study
Source: JMIR Form Res. 2025 Jul 31;9:e70146. doi: 10.2196/70146 (PMC12313082; doi:10.2196/70146)
Supplement: Multimedia Appendix 1 [file formative-v9-e70146-s001.pdf]

Appendix 1. Participant Interview Guide

For those who HAVE USED the portal and/or telehealth before / ARE REGULAR USERS

The purpose of today's interview is to ask for your feedback in understanding what you like and don't like about MyChart and telehealth. It doesn't matter whether you currently use MyChart or telehealth; we want to understand your experiences and your perspectives about these resources. We're hoping the information you share might help us learn how to we can improve these technologies for patients.

MYCHART

I noticed from your survey responses that you have used the patient portal, also called MyChart. To start, I'd like to talk more about your experiences using MyChart.

1. How did you first hear about MyChart?
2. Before you used MyChart, did you use any of the *[organization]* resources to help you learn about it?
    a. For example, had you read about it on the website?
    b. Watch the instructional video?
    c. Were you given any written instructions like a handout?
    d. Or did you use the telephone help line?
3. What were your first experiences like, for example, with logging in?
4. A lot of people have trouble remembering how to log in and navigate through MyChart. What strategies do you use to remember these things?
5. What would you like to be able to do with MyChart that you can't currently do?
6. Tell me more about what you like about MyChart. How does it help you manage your health?

Added subsection on test results:

7. Some people have mentioned that they like how they can view their test results quickly through MyChart. Has this been your experience?

    a. [If yes] Tell me about that – i.e., help me understand why you like getting your test results quickly versus waiting for your doctor to tell you your results.

    [If no] OK, tell me about your experience. *[Then ask appropriate follow-up questions based on what they say.]*

    b. [If yes] Do you see any downside to getting your test results quickly?

Some doctors have concerns about patients receiving their test results quickly. Specifically, doctors often don't have a chance to explain what the results truly mean, and they worry that patients' misinterpretations may cause emotional harm. This may especially be true if the results are about a cancer diagnosis or test result. What are your thoughts about this?

Note: a person with cancer may even disclose that this is how they learned about their cancer diagnosis. If this happens, keep pursuing this line of questioning. i.e., How did they feel? Did they wish things were different?

How would your feelings differ if we were talking about viewing test results that weren't life threatening, for example, a single A1C or blood sugar test result for diabetes?

Given the potential for misinterpretation, do you think some test results should NOT be released directly to patients through MyChart? (In other words, do you think some test results should only be communicated by doctors directly to patients, even if that takes a little longer?) Why do you think that?

TELEHEALTH

1. Now, I see that you've also completed at least one telehealth appointment, or video visit. Tell me about that.
2. About how many telehealth appointments have you completed?
    a. How did you first hear about the opportunity to do a video visit?
    b. Who offered it to you?
    c. What did they tell you about it and how it would work?
    d. Did they give you any instructions about how to connect?
3. Did you explore any of the resources for help with your telehealth appointment, like the instructions on the website or the telephone help line?
    a. A lot of people have trouble with their first telehealth appointment. Tell me about your first experience getting the telehealth appointment started and how you got through any challenges.
    b. Some people have a hard time with the fact that once you log in and are ready for the doctor to come on, there's kind of a "dark screen". What was your experience like?
    c. Tell me more about what you like about telehealth appointments. How do you feel like it helps you manage your health?
    d. What don't you like about telehealth? When is it not useful?

MYCHART AND TELEHEALTH

1. We think more people could benefit from using MyChart and telehealth, but they aren't aware that these resources exist. How could we make people more aware of MyChart and telehealth?
2. How would you describe MyChart to someone else?
3. How would you describe a telehealth appointment to someone else?
    a. How would you explain the pros and cons?
4. When it comes to using MyChart and telehealth, we've learned that different people need different levels of assistance. Help me brainstorm some ways to help people who have trouble using MyChart and telehealth.
    a. Probe: instructional brochures, online videos, live chat help, on-call navigator, in-house navigator, group classes (when/where would be ideal)
5. What kind of information would convince you to use MyChart or telehealth if you weren't currently a user?
    a. For example, would patient satisfaction scores be persuasive?
    b. What if we could report that patients using MyChart had better health outcomes, like A1C management or lower reported pain, than patients not on MyChart?

For those who HAVE NOT USED the portal and/or telehealth before / are NOT REGULAR USERS

We noticed from your survey responses that you do not use MyChart. That's OK.

MYCHART

Have you ever heard of MyChart?

IF YES – HEARD OF MYCHART:

1. What is MyChart, in your own words?
2. When and how did you first hear about it?
3. Have you ever explored any of the *[organization]* resources to help you learn about it?
    a. For example, have you read about it on the website?
    c. Watched the instructional video?
    d. Have you been given any written instructions like a handout?
    e. Have you used the telephone help line?
4. What are the reasons you are hesitant about using MyChart, or you have decided you aren't interested in using MyChart?
5. Is there anything *[organization]* could do to change your mind?
    a. For example, would it be helpful if someone walked you through the process in person or over the phone?
    b. Would you like to know how many other *[organization]* patients use MyChart?
    c. Would you like to know how using MyChart might improve your health?

Added subsection on care partner use:

6. Does anyone help you use MyChart?
    a. What does ___ help you do? That is, what functions does he/she use (e.g., appointments, contacting your doctor)?
    b. How often does ___ help you?
    c. What do you do when ___ is not available to help you?
    d. How do you feel about their using your account? For example, do you trust ___ to be honest with you about your health information and needs?

IF NO – NEVER HEARD OF MYCHART:

OK, since you're not familiar with MyChart, let's talk a little bit about what you do when you need help with different medical needs.

1. Currently, when you need an appointment, how do you get one? (e.g., call, make one at the previous appointment, go in person to make an appointment, wait to last minute and use ED)
2. When you are curious about your test results, what do you do? (e.g., call, wait to find out at the next appointment, assume it's OK unless they tell you otherwise)
3. When you need a medication refill, what do you do? (e.g., call your doctor, call the pharmacy, go in person to request one?)
4. When you have a medical question or need advice, what do you do? (e.g., call, go to urgent care, ask a friend?)
5. Would you be interested in trying MyChart with your own medical record if someone could help walk you through it and explain more about it?

TELEHEALTH

I noticed from your survey that you haven't used telehealth. Have you ever heard of telehealth, or video visit appointments?

IF YES, BUT NOT A REGULAR USER:

1. What have you heard?
2. Before your first telehealth appointment, did you explore any of the *[organization]* resources about it (e.g., the virtual care website or the telephone help line)?
3. What are the reasons you are hesitant about doing video visit appointments, or you have decided you aren't interested in video visits?
4. Is there anything *[organization]* could do to change your mind?
    a. For example, would it be helpful if someone walked you through the process in person or over the phone?
    b. Would you like to know how many other *[organization]* patients use telehealth?
    c. Would you like to know how using telehealth might improve your health?

IF NO:

Explain: Telehealth appointments are just like regular medical appointments with your doctor, but instead of them taking place in a doctor's office, you would use your smartphone or a computer to talk with the doctor over a live video. If you've ever done or FaceTime or Zoom, it's just like that.

1. As I describe telehealth to you, what are your concerns or questions about it?
2. Would you be interested in trying telehealth if someone could help walk you through it and explain more about it?
3. What do you think are some of the advantages of doing telehealth appointments?

OK to share: people who do telehealth say some of the advantages are: not having to drive to their appointment, which saves time and gas money; the convenience of being able to have their appointment from anywhere; and that it's great when you're feeling really lousy, or conversely, when it's just a super minor issue.

4. What do you think are some of the disadvantages of doing telehealth appointments?

OK to share: People who currently do telehealth say some of the disadvantages are: your provider can't do a physical exam on you, and some people feel it's more impersonal than seeing a provider in clinic. Overall, however, most people really like telehealth appointments, at least for some of their appointments when their doctors suggest that it may be appropriate.

5. For patients who are willing, we would like to offer help with setting up MyChart and telehealth on their devices so they can try out these technologies for themselves. We would like to support patients in whichever ways would be the most helpful, and we'd like your feedback about what that would look like. Please tell me which of these methods appeals to you and why.
    a. Step-by-step instructional brochure: (URL and handout provided)
    b. Step-by-step online videos: (URL provided)
    c. On-call phone help line: (phone number provided), Monday-Friday 8am-5pm
    d. Live text chat
    e. On-call navigator available in clinic
    f. Group classes available in clinic
