# Supplementary material for: Factors Associated With Portal and Telehealth Uptake and Use in a Minoritized, Low-Income Community: Mixed Methods Study
Source: JMIR Form Res. 2025 Jul 31;9:e70146. doi: 10.2196/70146 (PMC12313082; doi:10.2196/70146)
Supplement: Multimedia Appendix 2 [file formative-v9-e70146-s002.pdf]

Appendix 2. Interview Recruitment Consort Diagram

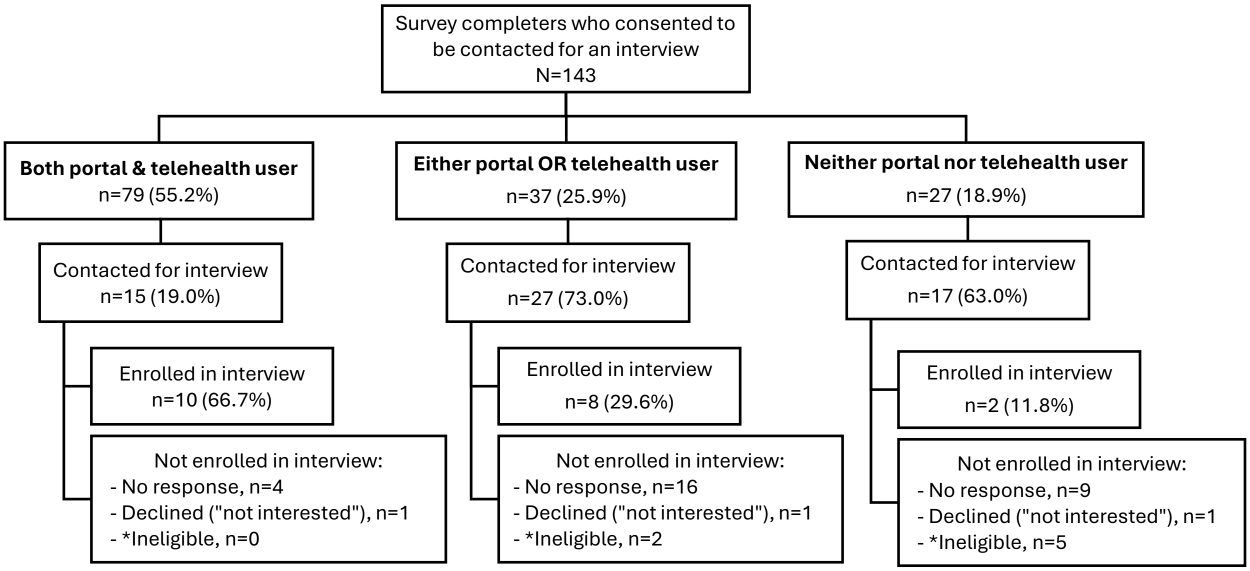

*At time of interview recruitment, participant did not have Wi-Fi and/or smartphone access.
