# Supplementary material for: Factors Associated With Portal and Telehealth Uptake and Use in a Minoritized, Low-Income Community: Mixed Methods Study
Source: JMIR Form Res. 2025 Jul 31;9:e70146. doi: 10.2196/70146 (PMC12313082; doi:10.2196/70146)
Supplement: Checklist 1 [file formative-v9-e70146-s003.pdf]

Standards for Reporting Qualitative Research (SRQR)

No.	Item	Description	Section
**Title and Abstract**			
1	Title	Concise description of the nature and topic of the study. Identifying the study as qualitative or indicating the approach (e.g., ethnography, grounded theory) or data collection methods (e.g., interview, focus group) is recommended	1, 2, 3
2	Abstract	Summary of key elements of the study using the abstract format of the intended publication; typically includes background, purpose, methods, results, and conclusions	2
**Introduction**			
3	Problem formulation	Description and significance of the problem/phenomenon studied; review of relevant theory and empirical work; problem statement	3
4	Purpose or research question	Purpose of the study and specific objectives or questions	3-4
**Methods**			
5	Qualitative approach and research paradigm	Qualitative approach (e.g., ethnography, grounded theory, case study, phenomenology, narrative research) and guiding theory if appropriate; identifying the research paradigm (e.g., postpositivist, constructivist/ interpretivist) is also recommended; rationale	4
6	Researcher characteristics and reflexivity	Researchers' characteristics that may influence the research, including personal attributes, qualifications/experience, relationship with participants, assumptions, and/or presuppositions; potential or actual interaction between researchers' characteristics and the research questions, approach, methods, results, and/or transferability	6
7	Context	Setting/site and salient contextual factors; rationale*	4
8	Sampling strategy	How and why research participants, documents, or events were selected; criteria for deciding when no further sampling was necessary (e.g., sampling saturation); rationale*	5-6
9	Ethical issues pertaining to human subjects	Documentation of approval by an appropriate ethics review board and participant consent, or explanation for lack thereof; other confidentiality and data security issues	6
10	Data collection methods	Types of data collected; details of data collection procedures including (as appropriate) start and stop dates of data collection and analysis, iterative process, triangulation of sources/methods, and modification of procedures in response to evolving study findings; rationale*	6
11	Data collection instruments and technologies	Description of instruments (e.g., interview guides, questionnaires) and devices (e.g., audio recorders) used for data collection; if/how the instrument(s) changed over the course of the study	5-6
12	Units of study	Number and relevant characteristics of participants, documents, or events included in the study; level of participation (could be reported in results)	7-8
13	Data processing	Methods for processing data prior to and during analysis, including transcription, data entry, data management and security, verification of data integrity, data coding, and anonymization/deidentification of excerpts	6
14	Data analysis	Process by which inferences, themes, etc., were identified and developed, including the researchers involved in data analysis; usually references a specific paradigm or approach; rationale*	6

Bridget C. O'Brien, Ilene B. Harris, Thomas J. Beckman, Darcy A. Reed, David A. Cook. Standards for Reporting Qualitative Research: A Synthesis of Recommendations. Academic Medicine, Volume 89, Issue 9, September 2014, Pages 1245-1251.

Standards for Reporting Qualitative Research (SRQR)

No.	Item	Description	Section
15	Techniques to enhance trustworthiness	Techniques to enhance trustworthiness and credibility of data analysis (e.g., member checking, audit trail, triangulation); rationale*	not performed; see PMID 27340178
16	Synthesis and interpretation	Main findings (e.g., interpretations, inferences, and themes); might include development of a theory or model, or integration with prior research or theory	11-14
17	Links to empirical data	Evidence (e.g., quotes, field notes, text excerpts, photographs) to substantiate analytic findings	11-14
**Discussion**			
18	Integration with prior work, implications, transferability, and contribution(s) to the field	Short summary of main findings; explanation of how findings and conclusions connect to, support, elaborate on, or challenge conclusions of earlier scholarship; discussion of scope of application/ generalizability; identification of unique contribution(s) to scholarship in a discipline or field	15-17
19	Limitations	Trustworthiness and limitations of findings	16-17
**Other**			
20	Conflicts of interest	Potential sources of influence or perceived influence on study conduct and conclusions; how these were managed	18
21	Funding	Sources of funding and other support; role of funders in data collection, interpretation, and reporting	17-18

*The rationale should briefly discuss the justification for choosing that theory, approach, method, or technique rather than other options available, the assumptions and limitations implicit in those choices, and how those choices influence study conclusions and transferability. As appropriate, the rationale for several items might be discussed together.

Bridget C. O'Brien, Ilene B. Harris, Thomas J. Beckman, Darcy A. Reed, David A. Cook. Standards for Reporting Qualitative Research: A Synthesis of Recommendations. Academic Medicine, Volume 89, Issue 9, September 2014, Pages 1245-1251.
